# Child malaria treatment decisions by mothers of children less than five years of age attending an outpatient clinic in south-west Nigeria: an application of the PEN-3 cultural model

**DOI:** 10.1186/1475-2875-9-354

**Published:** 2010-12-08

**Authors:** Juliet Iwelunmor, Oladipo Idris, Adeniyi Adelakun, Collins O Airhihenbuwa

**Affiliations:** 1 Department of Biobehavioral Health, The Pennsylvania State University, 315 Health and Human Development East, University Park, PA 16802, USA; 2 Department of Family Medicine, Lagos State University Teaching Hospital, Lagos, Nigeria

## Abstract

**Background:**

Using the PEN-3 cultural model, this study sought to understand mothers treatment decisions about their child febrile illness by examining positive health beliefs and practices held by mothers, examine existential (unique) practices that are indigenous to mothers and have no harmful health consequences, and explore negative beliefs and practices that limit recommended responses to febrile illness in children.

**Methods:**

This qualitative study was conducted in the paediatric section of an outpatient clinic in south-west Nigeria. A total of 123 mothers with children less than five years of age with febrile illness diagnosed as malaria by physicians were individually interviewed on their treatment-seeking practices prior to visiting the clinic and their reasons for attendance at the clinic.

**Results:**

For some mothers interviewed, effective treatment from the clinic for their child's febrile illness, coupled with physician's approach with malaria diagnosis and treatment practices was important in generating positive maternal treatment-seeking responses to child febrile illness. In addition, beliefs related to a child teething highlighted existential decisions with treatment-seeking for child febrile illness in this setting. Finally, the belief that febrile illness is not all that severe despite noticeable signs and symptoms was a concerning negative perception shared by some mothers in this study.

**Conclusion:**

The findings highlight the need to consider not only the responses that may serve as barriers to effective treatment, but also an acknowledgment of the positive and existential responses that are equally critical in influencing mothers' management of malaria in their children.

## Introduction

A particularly important question that malaria control programmes continue to ask is: "*why do some mothers continue to delay seeking help for their child's febrile illness, while others take their children promptly to health care centers within 24 hours after the onset of fever?" *[1]. As a result, empirical data from several malaria endemic countries [2-8] have supported the need for a deeper exploration of maternal perceptions and responses with child febrile illness presumed to be malaria as they are critical with the success of increasing treatment seeking for child malaria at health clinics. Indeed, it has been suggested that "mothers, regardless of their sociodemographic characteristics, make the first diagnosis of their child's febrile illness by defining and interpreting changes in their child's behaviour and temperature [7] Also, mothers are generally seen as the "first source of treatment"[3] as they often identify various treatment modalities and where to seek treatment for child malaria. While treatment costs and access to health clinics may influence patterns of treatment-seeking behaviour for child febrile illness [9-13], few qualitative studies have been conducted to explore how mothers respond to fever in their children, particularly in situations in which health care services are free. It is possible that features of the health care systems may encourage maternal use of recommended treatments for child febrile illness; however, relatively little attention has been given to understanding positive or unique decisions made by mothers about their child's febrile illness in the context of free health care services.

Alongside the calls for increased understanding of maternal practices that may improve the quality of malaria case management [14], there is a need to also highlight positive health beliefs and practices held by mothers that are beneficial for malaria control-existential (or unique) practices that have no harmful health consequences-rather than focusing only on negative perceptions or beliefs related to child malaria. Also, in the context of treatment-seeking behaviours, there is recognition that treatment decisions reflect shared knowledge and experiences that are embedded in local culture [14-17]. As a result, in many malaria-endemic countries particularly in sub-Saharan Africa, it is not uncommon for people to engage in multiple patterns of treatment that are customary and may or may not correspond with biomedical standards [18,19]. For some mothers, it is possible that treatment decisions may arise out of unique cultural beliefs and practices that have no harmful health consequences. As noted by Williams and Jones [18], "*instead of trying to provide an answer to the question how can we get them to ..., we should be pressing to find ways to increase people's capacity to access and complete effective treatments*". The need to improve people's capacity is critical with the success of malaria control initiatives. In this paper, such efforts are presented using the PEN-3 cultural model.

### Theoretical framework: The PEN-3 cultural model

**Figure 1:**
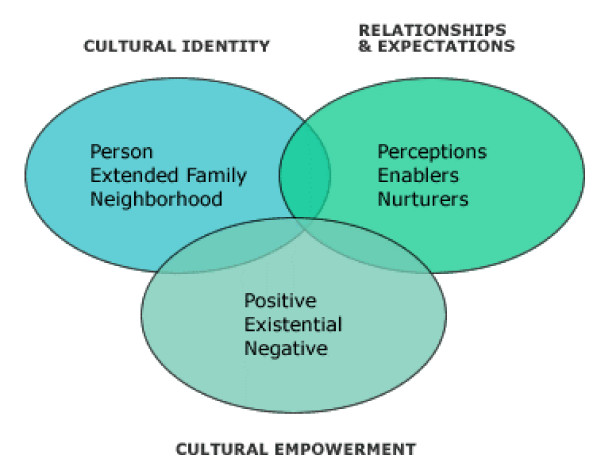
**The PEN-3 cultural model highlights the impact of a behaviour on health (positive, existential or negative), the key influences of the behaviour (perceptions, enablers, or nurturers) and the focus of the health behaviour intervention (person, extended family, or neighbourhood)**.

Developed by Airhihenbuwa [17,20,21], the PEN-3 cultural model aims to address the complexity of health issues by addressing cultural beliefs and practices that are critical to health behaviours and should either be encouraged, acknowledged, and/or discouraged. The PEN-3 cultural model (see Figure 1) contextualizes the role of culture in shaping understanding of and actions towards health and illness. It consists of three dimensions that are dynamically interrelated and interdependent: Relationships and Expectations, Cultural Empowerment, and Cultural Identity [20].

Of particular interest to this study is the cultural empowerment domain, which explores the positive, existential, and negative aspects of behaviours of interest. While the positive aspects include values and relationships that promote the health behaviour of interest, the existential examines the qualities of behaviour that make it unique [17,20,21]. The negative aspects include health beliefs and actions that are harmful to health and should be changed [17,20,21]. In utilizing the cultural empowerment domain, this study identified mothers' positive decisions and practices related to treatment-seeking for their child's febrile illness, existential (unique) illness-related decisions that are important with the management of child febrile illness, and negative decisions that serve as barriers to appropriate treatment practices.

## Methods

### Setting

This study was conducted from July to September 2009, in an outpatient clinic in Lagos, located in the south-west region of Nigeria. This region is highly endemic for malaria, particularly during the rainy season (April-October) and an ideal location for understanding the factors that influence mother's perceptions of child malaria and patterns of treatment in the event of malaria. The outpatient clinic also provides free health services and antimalaria drugs for children less than five years of age.

### Data collection

Qualitative data collection methods consisting of individual in-depth interviews were conducted with mothers of children less than five years of age attending the clinic. A purposive sampling approach was used to recruit only mothers of children with febrile illness diagnosed by physicians as malaria. A total of 123 consenting mothers participated in this study and they were individually interviewed on their treatment-seeking practices prior to visiting the clinic. Participants were informed of the objectives of the study prior to the start of the interviews. Each interview took about 20-30 minutes to complete and mothers were interviewed alone without any input from family members who had accompanied them to the clinic. Ethics approval was granted by Penn State and the Lagos State University Teaching Hospital.

### Data analysis

The in-depth interviews were analyzed using the principles of content analysis described by Morse and Field [22]. Each interview question was reviewed so as to identify different salient themes in the content of the interview. Specifically, following the initial review, the interview questions were segmented into several important topics, which became the primary categories. Data were then sorted into each category until saturation was reached or until no new category emerged. Once the categories had ample data, the PEN-3 cultural model was used as a guide to organize the categories into themes by looking for relationships between and within each category that identified aspects of maternal responses to child febrile illness subsequently diagnosed as malaria by physicians that represented positive, existential (unique), or negative response to treatment-seeking practices.

## Results

Table 1 summarizes the demographics of the participants in the study. Also, using the PEN-3 cultural model, the results were grouped into three main themes that emerged from the in-depth interviews. The themes were labelled as: my child will get well (positive responses to child malaria), teething caused my child's fever (existential responses to child malaria), my child's illness is not severe (negative responses to child malaria).

**Table 1:** Characteristics of study population

Characteristic	Number (%)
**Ethnicity**	
Yoruba	69 (56.1)
Igbo	38(30.9)
Hausa	3(2.4)
Other	13(10.6)

**Mean number of children**	2.04

**Age (y)**	
Mean (range)	30.7 (20--43)

**Education**	
None	1(.8)
Primary	8(6.6)
Secondary	49(40.2)
Higher	64(52.5)

### "My child will get well": Positive responses to child malaria

Even though malaria remains a major cause of child morbidity and mortality among children and its signs and symptoms are critical in recognizing the onset of malaria, almost all of the mothers interviewed in this study believed that *"their children will get better after they see a doctor." *Although some of the mothers had given some form of treatment to their children prior to visiting the clinic as observed in other studies conducted in Nigeria [23,24], several statements, such as *"doctors' treatment will make child better*," and "*my child will be okay after medicine from clinic*," highlight some of the positive responses to treatment-seeking for child malaria at the clinic. Two important notions reflected in these responses are; the role of appropriate malaria treatment and the role of physician's in strengthening maternal capacity to positively respond to child malaria.

Mothers appeared to be well aware that proper malaria treatment at health clinics is critical to their children getting well. This was expressed by statements such as, "*by bringing child to hospital, I will receive something good to stop symptom*." Several mothers noted that although the *"persistence of symptoms" *was one of the chief reasons for bringing their children to the clinic, "*it was better to come so that the doctor will tell me why my child is ill*," and "*prescribe drugs that will make my child better*," and "*stop the symptoms*."

The notion that physician's treatment will "*help the child to get better*" was a sentiment shared by a several mothers interviewed in this study. Some mothers believed that physicians have the "*final say*" on treatments that will make a child better as they are "*specialized on children's illness*," and "*anything they give will be the best*." Also, mothers expressed that they did not "*mind waiting long hours to see a doctor*," as their *"services are the excellent*," and the "*drugs written by the doctor's are the best*." Nearly all the mothers interviewed valued the antimalaria drugs prescribed by the physician's rather than those bought from pharmacies. Many believed that the drugs prescribed by doctors are "*the right drugs*" needed to treat malaria as oppose to "*fake drugs*" found in many pharmacies in this setting and that these drugs will help their children's "*illness to go away*," Also, our findings indicated that "*doctor's approach*" to mothers was also critical in generating positive responses to child malaria, as one mother noted that *"they have the ability to put mothers at ease by explaining what is wrong*" and by giving "*the right drugs*" to " *help child to become better*."

### "Teething caused my child's fever": Existential (unique) responses to child malaria

To understand the cultural context of child febrile illness, mothers were asked to describe in their own words what they believed triggered their child's illness. The findings indicated that mother's perceptions of their child's febrile illness differed from those of the physicians in several existential ways. For example, when mothers were asked questions about what caused their children's febrile illness, regardless of the malaria diagnosis by physicians, to some of the mothers (34%), their children's illness was caused by teething. In aligning with the existential principles of the cultural empowerment domain, febrile illness perceptions related to teething are values and beliefs held by mothers in this setting that have no harmful health consequences in that they did not deter decisions to seek effective treatment at the clinic. Follow-up questioning revealed some of the reasons why mothers associated their child illness to teething. For example, one mother stated that she noticed that her child had "*swollen gums*" and another stated that her child was "*biting her gums*" and as a result the illness was triggered by teething. Also, the presence of child fever (high body temperature) was widely understood to be caused by teething, a view that arose largely through interactions with other family members who told some of the mothers that "*the eruption of a child's tooth "triggers the child's temperature*." Others stated that "*teething is responsible for a host of symptoms." *For example, one mother explained that "*high temperature and diarrhea, particularly blood in stool and loss of appetite*" were associated with teething. The belief that teething is responsible for high temperatures (fevers) prompted mothers to seek help at the health clinic (existential decisions) as they were of the opinion that treatment from the clinic would enable children to become better.

### "My child's illness is not severe": Negative responses to child malaria

Since malaria is often viewed as an "*ordinary illness*" in many endemic countries [1,9], of particular interest in this study was the way in which mothers perceived the severity of their child's illness. Evidence from this study indicated that 61 (52.1%) mothers did not consider their child's illness to be severe. This was particularly evident in responses such as "*my child's illness is mild*," and "*my child's illness is not all that severe*." Even in situations in which mothers stated that their children's illness was caused by *"malaria" *or "*exposure to mosquito bites*" and their children were experiencing noticeable signs and symptoms such as "*high temperature*," some still stated that their children's illness was "*not too severe*" and that it was "*manageable*." A similar phenomenon was found in Kenya [3,25] and Tanzania [1] where child febrile illness is often not perceived as severe but rather as a mild, ordinary illness. These perceptions have implications for malaria treatment practices as it often leads to delay in seeking prompt diagnosis and appropriate treatment at health facilities. In elaborating perceptions of perceived severity of illness, follow-up questioning revealed that some mothers believed that since their children were still "*active*" and "*playing around*", their children's illness were not severe. Indeed, studies have shown that caregivers have their own way of categorizing child fever into mild and severe illness [3,26]. For example, in describing the predictors of health-seeking behaviour relating to child fever among caretakers in Malawi, Chibwana and colleagues [26] found that caregivers believed that children with fever who were able to play were classified as having mild fever, while children with fever who could not play were considered as having severe fever. Mother's perceptions about the severity of their child's fever dictated their course of action with treatment as those who perceived the illness to be mild or not severe only sought effective treatment because the "*symptoms persisted*" and they wanted "*proper treatment*" that will make their "*child feel better*."

## Discussion

This study represents the first application of the PEN-3 cultural model with malaria treatment practices in endemic countries. The findings indicate that appropriate treatment from the clinic coupled with physician's approach with child malaria diagnosis and treatment were important in generating positive maternal responses to treatment seeking for child febrile illness. In addition, beliefs related to teething patterns were critical in revealing existential decision-making towards treatment seeking for child febrile illness in this setting. For example, even though some mothers were of the opinion that their child's febrile illness was caused by teething, they still utilized health care facilities for diagnosing and appropriate case management of illness. The notion that teething is part of a child's development process is an existential belief held by mothers in this setting that has no harmful consequence particularly as it posed no threat to maternal treatment seeking for child febrile illness. Also, there are negative responses that should be taken into consideration in formulating malaria control strategies. Consistent with previous studies [1,3,25], the belief that febrile illness is not severe was a common perception shared by some mothers in this study. This perception often underestimates the potential harm of child febrile illness and may invariably contribute to the estimated increase in child morbidity and mortality rates due to malaria in many endemic countries. Ultimately, the decision to seek prompt diagnosis and effective treatment may be influenced by whether mothers perceived the illness to be mild or severe. In cases in which child febrile illness was perceived as mild, or not severe, it was not uncommon for mothers to delay seeking treatment. As signs and symptoms became more prominent, mothers then sought care from health care facilities for proper treatment of their children's illness.

Although it remains unclear why some mothers underestimate the potential severity of child febrile illness, one might expect that if mothers have the ability to recognize signs and symptoms of malaria, they will act accordingly by providing appropriate anti-malarial treatment or by seeking a health care facility for prompt clinical examination. This was generally not the case in this study, for even in situations in which mothers perceived that their children's illness was caused by mosquito bites, they also stated that the illness was not severe. These negative responses suggests that knowledge of the causes of malaria or even signs and symptoms alone may be insufficient if efforts are not equally made to address perceptions related to illness severity for some mothers. Thus, the assumption that changing knowledge may lead to behavior change may be severely limiting if it fails to also consider other factors, including positive or existential factors that might influence treatment-seeking behaviours for child malaria. The PEN-3 cultural model offers an opportunity to explore not only the responses that may serve as barriers to effective treatment of malaria, but also positive and existential responses that are critical in influencing mothers' management of malaria in their children. Also, the findings clearly illustrate the importance of highlighting responses that promote treatment-seeking behaviours as well as responses that have no harmful consequences prior to identifying responses that may have negative health consequences. In this way, rather than dismissing the values and practices that mothers may have towards malaria treatment strategies, the PEN-3 cultural model affirms the possibilities of their lived experiences by encouraging responses that are positive, acknowledging unique responses while discouraging responses known to be harmful to health.

The implications of these findings are important for malaria control strategies. Specifically, as mothers adapt to the new and expensive artemisinin-based combination therapies with their multiday/dosage regimes [27], attempts to conceptualize the positive, existential, and negative factors that influence patterns of treatment-seeking for child fever are critical to framing a comprehensive approach to malaria treatment in endemic regions. The findings from this study should be considered in light of several limitations. First, the in-depth interviews were conducted among mothers who were recruited from a health care facility and the information obtained was based on interview responses that may be prone to bias. Second, the findings cannot be generalized to other mothers as the sample for this study was not randomly selected. Indeed, the degree of representation is unknown, particularly as we did not conduct interviews with mothers who did not bring their children to the outpatient clinic. Despite these limitations, the findings provide a better understanding of the influence of cultural values and practices with malaria treatment strategies in that it illuminates maternal response to child febrile illness that are positive, existential, or negative. Although the potential for sampling bias exists in this study, the use of purposive sampling ensured that only mothers with child febrile illness diagnosed as malaria were interviewed. Also, the similarities between maternal responses from one in-depth interview to another, coupled with consistency of findings with published studies on maternal perceptions and treatment-seeking practices for malaria, permits confidence in the validity and analysis of the data [28]. However, more research is necessary to assess other beliefs and values that influence decision-making for treatment seeking practices at clinic settings, the resources and institutional arrangements that promote or discourage prompt and effective treatment of malaria, coupled with the role of family, kin, and friends in influencing decisions related to patterns of treatment-seeking. These factors, which coincide with the relationship and expectations domain of the PEN-3 cultural model, should by explored further in order to achieve a deeper understanding of not only the social and cultural factors, but also the structural factors that might influence positive, unique, or negative responses to patterns of treatment-seeking for child febrile illness in malaria-endemic countries.

## Competing interests

The authors declare that they have no competing interests.

## Authors' contributions

JI designed the study, collected and analyzed the data and wrote the paper. OO and AA collected and analyzed some of the data and commented on the findings. COA analyzed some of the data, discussed the findings, and commented on the written paper. All authors read and approved the final manuscript
